# WAMI: a web server for the analysis of minisatellite maps

**DOI:** 10.1186/1471-2148-10-167

**Published:** 2010-06-06

**Authors:** Mohamed Abouelhoda, Mohamed El-Kalioby, Robert Giegerich

**Affiliations:** 1 Center for Informatics Sciences, Nile University, Giza, Egypt; 2 Systems and Biomedical Engineering Department, Faculty of Engineering, Cairo University, Giza, Egypt; 3 Practical Computer Science Department, Faculty of Technology, Bielefeld University, Bielefeld, Germany

## Abstract

**Background:**

Minisatellites are genomic loci composed of tandem arrays of short repetitive DNA segments. A minisatellite map is a sequence of symbols that represents the tandem repeat array such that the set of symbols is in one-to-one correspondence with the set of distinct repeats. Due to variations in repeat type and organization as well as copy number, the minisatellite maps have been widely used in forensic and population studies. In either domain, researchers need to compare the set of maps to each other, to build phylogenetic trees, to spot structural variations, and to study duplication dynamics. Efficient algorithms for these tasks are required to carry them out reliably and in reasonable time.

**Results:**

In this paper we present WAMI, a web-server for the analysis of minisatellite maps. It performs the above mentioned computational tasks using efficient algorithms that take the model of map evolution into account. The WAMI interface is easy to use and the results of each analysis task are visualized.

**Conclusions:**

To the best of our knowledge, WAMI is the first server providing all these computational facilities to the minisatellite community. The WAMI web-interface and the source code of the underlying programs are available at http://www.nubios.nileu.edu.eg/tools/wami.

## Background

### Minisatellite maps

**Figure 1:**
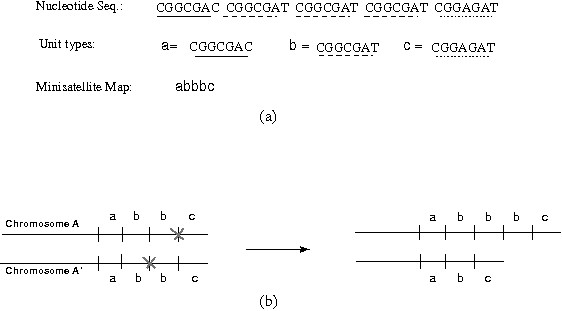
**A minisatellite map**. Part (a): A minisatellite locus and the respective map. The locus contains five units classified into three distinct unit types denoted by the symbols a, b, and c. Part (b): The unequal cross over causes duplication of unit b leading to the map in Part (a) of this figure.

A DNA region is categorized as a *minisatellite locus *if it is composed of tandemly repeated DNA stretches and spans more than 500 bp. Each of these stretches is called a *unit *and it holds (by most definitions) 10-100 bp. The units are not necessarily identical due to point mutations, and their number and organization may vary among individuals as a result of subsequent evolutionary events. The variant repeat mapping by PCR (MVR-PCR) is a popular technique to reveal the structure of a minisatellite locus as it enables unit typing and minisatellite map production. *Unit typing *is the classification of the variable units into distinct types (called variants and denoted by different symbols) according to their DNA sequences. A *minisatellite map *is a compact representation of the minisatellite locus, where each unit is replaced with the respective symbol. Figure 1(a) shows an example of a minisatellite locus and the respective map.

### Applications of minisatellite map analysis

Minisatellite maps have manifold applications in forensics and population studies. Foster et al. [1] used minisatellite maps to resolve the dispute on the fatherhood of President Jefferson to a son of his slave. They showed that Jefferson is the biological father of her last son, but not her first son as thought before. Based on the MS205 dataset, Armour et al. [2] confirmed the African origin for modern humans, Alonso et al. [3] proved a European affiliation for the Basques, and Rogers et al. [4] dated the Eurasian population as 52000-66000 years and the oldest European as 37600-56200 years. Using the MSY1 dataset, which was first investigated by Jobling et al. [5], Brión et al. [6] showed that European lineages are more similar than North African ones. Bonhomme et al. [7] used minisatellites to study house mouse population and provided a migration map for them. Very recently, Yuan et al. [8] used the MS32 minisatellites to study the population specificity among Thai, Chinese, and Japanese. They showed that the MS32 minisatellite is an effective tool to distinguish individuals from these populations.

The functional and medical roles of minisatellites have also been addressed in many studies for the last two decades, and the interest increases with more individual genomes becoming available. To mention a few examples, Thierry et al. [9] discovered a class of minisatellites involved in cell adhesion and pathogenicity. Vafiadis et al. [10] proved that the Insulin minisatellite plays an important role in the regulation of Insulin and the authors of [11,12] showed that it is associated with polycystic ovary syndrome, obesity, and type I diabetes. Raeder et al. [13] showed that the mutations in the CEL minisatellite is correlated with exocrine dysfunction in diabetic patients. Tsuge et al. conjectured that polymorphisms in minisatellites at the flanking region of SMYD3 are susceptible risk factors for human cancer [14]. For more studies, we refer the reader to the review of Vergnaud and Denoeud [15] and the WikiGenes page in [16].

### Computational challenges in minisatellite analysis

Researchers analyzing minisatellite maps usually perform the following computational tasks:

1. Comparison of minisatellite maps by computing all pairwise alignments.

2. Construction of a phylogenetic tree based on all pairwise distances, to show the relatedness between the involved individuals.

3. Studying structural variations, to examine how the unit types vary and distribute along a minisatellite map.

4. Studying duplication dynamics, to infer the type from which the map originated and in which direction the map elongates.

Recent studies often relied either on visual inspection or on heuristic methods. To our surprise, most did not make use of the recent advancement in the bioinformatics methods developed for pairwise map comparison [17,18]. We think this situation is mainly due to the lack of both web-servers and open source tools performing the aforementioned tasks. To the best of our knowledge, there is currently just one server, called MS_ALIGN (http://www.atgc-montpellier.fr/ms_align/) for minisatellite map comparison [17]. It is, however, limited to computing all pair-wise alignments, with no post-processing and visualization of map alignments.

In this paper, we present the web server WAMI for the analysis of minisatellite maps. The server uses a recent algorithm for map alignment, improved over the one in MS_ALIGN, and provides a workflow for the execution of the four computational tasks mentioned above, including visualization. These capabilities are demonstrated here by the analysis of the MSY1 [19] and MS205 [2-4,20] datasets.

## Implementation

### Model of minisatellite map evolution and alignment

Minisatellite maps can be studied in an independent or a comparative fashion. In the former, a map is analyzed to identify the evolutionary history that gave rise to the observed sequence of units. In the latter, two maps are aligned together to figure out regions of common and individual evolution histories. However, both tasks are entangled, since a region of individual evolution, juxtaposed to a gap in the map alignment, must have a plausible individual history. This makes minisatellite map alignment algorithmically more challenging than the standard sequence alignment problem.

#### Map evolution

Our evolutionary model of minisatellite maps includes the following operations acting on the unit level:

• *Unit mutation*: This is the change of one unit type into another. For example, the unit b in the map abd mutates into c leading to the map acd. The unit mutation is a consequence of point mutations (substitution and indels) acting on the DNA sequence of the units. In the example of Figure 1, the differences between the three unit types are attributed to nucleotide substitutions.

• *Duplication*: Duplication (also known as expansion or amplification) is the generation of new copies of the units by tandem duplications. Replication slippage, reciprocal exchange (unequal crossover or unequal sister chromatid exchange), and gene conversion (including synthesis-dependent strand annealing, abbreviated by *SDSA) *are potential mechanisms for unit duplications. The first is suggested for short segments while the others are for long ones; see [21-25] for more details. Figure 1(b) illustrates the unequal cross over mechanism, where the paired homologous chromosomes exchange unequal segments during the cell division. This results in the duplication of the unit b in one chromosome and the deletion (contraction) of it in the other. The *single-copy *duplication model assumes that one unit can duplicate at a time while the *multiple-copy *duplication model assumes that multiple adjacent units can duplicate at a time. For example, the adjacent units bc in the map abbc can duplicate in one event, leading to the map abbcbc.

• *Insertion/Deletion*: Insertion is the appearance of unit types, possibly due to errors or translocation events. For example, insertion of unit z in the map ac leads to the map azc. A dual operation to insertion is deletion where one unit disappears, leading also to map contraction. Potential mechanisms for these events include the ones mentioned above except for replication slippage.

Each of these operations is assigned a cost to reflect the relative rate at which it occurs in nature. The cost of a unit mutation is proportional to the Hamming/edit distance between the nucleotide sequences of the units. We write *d*_*M*_(*x*,*y*) to denote this cost between two units *x *and *y*. (Of course, *d*_*M*_(*x*,*y*) = 0 if *x *= *y*.) In Figure 1, *d*_*M*_(a,b) = 1 because of one mismatch at the last nucleotide, and *d*_*M*_(a,c) = 2 because of mismatches at the fourth and the last nucleotide. The costs of duplication, insertion, and deletion are arbitrary and usually chosen such that the duplication is less than the mutation, deletion and insertion cost.

#### Reconstruction of evolutionary history

The *evolutionary history *of a map is the series of evolutionary operations leading to the observed sequence of units. This history is also called *duplication history*, because the duplication is the main event contributing to map evolution. The cost of a duplication history is the total cost of the occurring operations. An *optimal *(most parsimonious) history is one with the minimal cost. For example, one history of the map bcaccbb originated from the leftmost unit b is as follows: The leftmost unit b duplicated three times to the right leading to the sub-map bbbb. Then the second b mutates into c leading to the sub-map bcbb. The unit c duplicates two times to the right producing the sub-map bcccbb. The second c mutates into a and the last c duplicates once again to the right leading to the final observed map. Assuming that *d*_*M*_(a,b) = *d*_*M*_(b,c) and *d*_*M*_(a,b) <*d*_*M*_(a,c), we leave it as an exercise for the reader to verify that this scenario is indeed an optimal one.

#### Map alignment

The alignment of the minisatellite maps includes the operations of replacement (match/mismatch, where mismatch corresponding to mutation), free insertion/deletion (indel), and duplication. Given a cost for each operation, an optimal alignment is the one of minimum cost. An efficient algorithm for finding optimal map alignments is ARLEM [18].

**Figure 2:**
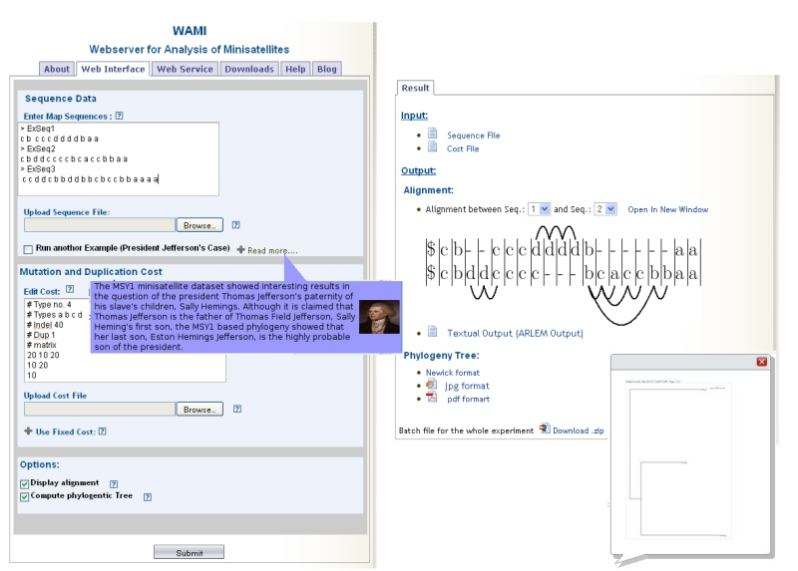
**WAMI main web-interface**. The upper sections include input of maps and cost file as well as some examples. A description of the example of the President Jefferson's dataset is shown. The lower section sets the option for building a phylogenetic tree over the input map. Note that there are other related tabbed pages, including introduction, web-service, download, and help/Blog pages. Right: The result page of WAMI. The output is organized into three categories: Alignment, phylogeny, and batch retrieval. In the alignment category, all pairwise alignments can be displayed. Here, an alignment between maps one and two (given in the left screen shot) is visualized. The replaced (match/mismatch) units are put above each other. An arc connecting two *identical *units corresponds to a duplication event, and an arc connecting two *different *units corresponds to a duplication followed by a mutation events. The sub-map composed of the units " bcaccbb" of the lower sequence emerged from the leftmost unit b of this sub-map. The duplication history was the one explained in the subsection about duplication history and alignment model. The category showing the phylogenetic tree appears only if this option was set. We provide the tree in text, JPEG (shown image), and PDF format. Finally, we provide a link to a compressed file containing all the input/output files of a WAMI run.

An optimal map alignment delivers a three-stage scenario: The aligned units (match/mismatch) refer to common ancestors, the duplications refer to differences in the individual duplication histories, and the indels may refer to errors or translocations. Figure 2 (right), shows an alignment of two maps where the replaced (matched/mismatched) characters are put above each other and the units evolved by duplications are attached to arcs. In this representation, an arc connecting two *identical *units corresponds to a duplication event, and an arc connecting two *different *units corresponds to a duplication followed by a mutation. In this alignment, the sub-map bcaccbb has emerged as a result of duplication/mutation events from the leftmost unit b. This sub-map is the example given above in the duplication history, and no indels exist.

Biologically, the map alignment, compared to individual map analysis, provides clues about the timing and direction of map evolution as well as the type of operation. In the alignment, we can conclude that the replaced units had appeared before the units evolved by duplications because of inheritance. We can also conclude that the evolved regions emerged from the inherited units that occur either on its left or right side. Furthermore, if we know that one sequence is the ancestor of the other, then we can distinguish better between the loss and gain of units, i.e. contraction versus duplication and insertion versus deletion.

#### Extension beyond the single-copy model

For WAMI, we extended ARLEM with a simple heuristic algorithm to account for double-copy duplications, where at most two different units can duplicate at a time, e.g., bc→bcbc. The idea of our algorithm is to pre-process the map to locate each sub-array of units of the form "xyxy...", where x ≠ y. We then create a new type X = xy and replace the units in this array with the new type to yield the array "XX...". The distance between the new type X and each original type z is the cost of optimal duplication history of xy emerged from or contracted to z. The distance between two new types X = xy and X' = x'y' is the cost of aligning xy and x'y'. Finally, the alignment algorithm of ARLEM runs on the transformed map using the new distances between the map units. In WAMI, the use of double copy model is optional, because the single copy duplication model is already sufficient for many data sets.

Computationally, it is infeasible to infer a history under the still more general, multiple copy duplication, model involving arbitrary number of copies [26].

### Four tasks supported by WAMI

#### Fast computation of pairwise map alignment

The basic step in WAMI is the computation of all pairwise alignments of the input maps, uploaded or edited online in multi-fasta format. The user can use default parameters (costs of each operation) or specify other ones through the use of a cost file uploaded to the server.

The map alignment model implemented by ARLEM allows that aligned units duplicate either to the left or to the right. For example, the sub-map dd in the aligned lower sequence of Figure 2 (right) was originated from the inherited unit c on the right, where c duplicated to the left to produce cc, then the leftmost c mutated into d which eventually duplicated to the left to produce another d. Previous programs allowed duplication only in left-to-right direction, where such a scenario cannot be modeled. This leads to an alignment of higher cost. This symmetric feature is crucial for studying the direction of map elongation, discussed below.

#### Phylogenetic tree construction

WAMI uses the program BIONJ [27] to construct the phylogenetic tree from the pairwise distances computed by ARLEM. BIONJ is based on a neighbor joining algorithm. The program njplot [28] is then used to visualize the tree.

#### Analysis of structural variation

In studying structural variation, researchers try to identify highly variable regions of the map. Most previous studies showed that map extremities are more variable than other map regions, a phenomenon known as (bi)polar variability [2,5,20]. WAMI can automatically provide evidence of (bi)polarized variation for a given dataset based on a scramble (randomization) test.

The program ARLEM was augmented with an extra option that determines the location associated with half of the optimal score in the aligned maps. We call this location the *pivot-point*. The rationale of the pivot point is that if the variations were accumulated at one end, then the pivot-point would be shifted towards this end. The pivot-points are calculated for all pairwise alignments and normalized with respect to the respective sequence lengths. A histogram for the pivot-points is then generated. To qualify the results, WAMI computes another histogram for a randomized dataset obtained by shuffling the units in each map of the input dataset. It is expected that the histogram for uniformly distributed unit types along the maps is close to the Gaussian distribution, centered around the value 0.5. WAMI produces a single plot containing the two histograms overlaid on each other. The results section contains examples of applying this procedure to MS205 and MSY1 datasets.

#### Analysis of duplication dynamics

Determining the direction in which the units duplicate is an interesting issue that can help in inferring the evolutionary processes and the source/origin unit of the map. For the MSY1 dataset, for example, Jobling et al. [5] conjectured that Type 4 (4a) is the source of the map and assumed that the units preferably duplicate in the 3' →' 5' direction. WAMI has a procedure that can test this kind of hypothesis based on another scramble test.

In ARLEM, units are allowed to duplicate towards the left or towards the right to achieve the best alignment score, while accommodating the most parsimonious series of duplication events. We added an option to ARLEM to restrict the duplications to originate either from the leftmost or from the rightmost unit of a map interval with duplication events. For example, if only the option imposing left-to-right duplication origin was set, then the sub-map "dd" in the aligned lower sequence of Figure 2 (right) could not have been originated from the unit "c" on its right, leading probably to increased alignment cost under this restriction.

To detect directional bias, WAMI invokes ARLEM three times on the dataset: 1) with both duplication directions allowed, 2) with only left-to-right duplications allowed, and 3) with only right-to-left duplications allowed. The latter two cases tend to yield higher cost than the first, because the duplications may be forced to follow a non-parsimonious scenario. Then the number of alignments in the second invocation with cost higher than the optimal one (as determined by the first invocation) is counted. Let *E*_*l *_denote this number. The analogous number *E*_*r *_for the third invocation is also computed. A normalized value combining both figures *E*_*n *_= (*E*_*l *_- *E*_*r*_)/*E*_*l *_+ *E*_*r*_) is then computed. The idea is that if *E*_*l *_was different from *E*_*r*_, and *E*_*r *_was small, then *E*_*n *_would converge to + 1, and one could argue that the duplications in the right-to-left direction are almost sufficient to yield alignments close to the optimal ones. Hence, right-to-left duplications appear preferred in the evolution of the minisatellites at hand. To further validate the results, WAMI runs a scramble test and computes the normalized *E*_*n *_values for many random datasets, obtained by shuffling the map units. Finally, the *E*_*n *_values are summarized in a histogram and plotted along with a peak representing *E*_*n *_of the original dataset. For random datasets where duplications to the left and to the right occur in an equal rate, it is expected that the distribution of *E*_*n *_is close to the Gaussian distribution centered around the value zero.

The scramble test is compute intensive, because the map alignment phase is repeated many times over scrambled datasets of the same size as the original. To speed up the computation, we use an approximation technique. We reduce each map to its *modular structure*, which is the sequence of distinct units in the map. For example, the modular structure of the map aaabbc is abc. This is reasonable because transitions between unit types strongly indicate the direction of duplication. Because the modular structure is typically much shorter than the map, a significant speed up is achieved.

### User Interface

WAMI has an easy to use and intuitive interface. The main web-page contains four examples to help the user format map data and cost file. (One example is about the real dataset for the President Jefferson's fatherhood case, mentioned above. Other two examples about some published maps of the MSY1 [4] and MS205 [2-4,20]. datasets.) Tool tips and a help menu are also provided. For sustainability of service, we attached a blog to the web-site, to collect user feedback and learn about new features requested by the community. A part of the main interface is shown in Figure 2 (left).

Upon job termination, the user is directed to the results page, where pairwise alignments are displayed and one can toggle between them, see Figure 2 (right). The duplication events within optimal alignments are represented by arcs. The images depicting the alignments are produced based on LaTeX. (The respective Tex files are included in the batch download). If the phylogeny option was chosen, the tree in Newick/JPEG/PDF format can be retrieved. The results of structural variations and duplication dynamics options are summarized and presented to the user in the form of histograms. For datasets larger than 50 sequences, the user is prompted to enter an email address to receive a notification when the job terminates. All these results can be downloaded as a compressed file.

### Computational efficiency

The program ARLEM uses a highly optimized algorithm for map alignment. It is based on a compression technique to save redundant computations and its speed is not affected by any increase in the number of types. In [18], we reported that ARLEM is 18 to 24 times faster than the previously available algorithm MS_ALIGN, using real and artificial datasets. For further speed-up, the options for computing phylogeny, analyzing structure variations, and duplication dynamics run in parallel over a computer cluster of four nodes, where each node contains two Quadcore CPUs (2.5 GHz each) with 64 GB RAM.

## Results and Discussion

The examples given in the sequel are based on the minisatellite datasets MSY1 [5] and MS205 [2-4,20]. The former dataset is composed of 345 maps and the number of distinct unit types is eight; the types are assigned the codes {0, 1, 1a, 2, 3, 3a, 4, 4a}. The latter dataset is composed of 653 maps of which 429 valid maps belong to haplotype C [4]. The number of distinct unit types is two and the types are assigned the codes {A,T}.

### Alignment and phylogeny

Table 1 shows the running times for real and artificial datasets of varying sizes and for different scramble test parameters. The number of iterations is the number of random datasets analyzed for studying the directional bias based on the modular map structure. The number of iterations based on the non-modular structure is a multiplication of the alignment time. The time for constructing the phylogenetic trees is not shown in the table, because it is in the range of seconds, i.e., negligible compared to other steps. The alignment time of the MSY1 dataset is higher than that of the MS205 because the average length of the MSY1 maps is higher than that of the MS205. But in analysis of directional bias, MS205 takes more time because the average length of its modular structure is three times the one of MSY1 with much higher variability, and our approximation technique described above is less effective for MS205. (The average modular structure lengths is approximately 13 and 4 for the MS205 and MSY1, respectively.) The random datasets were generated such that each map has an average length of 80 units (minimum and maximum are 60 and 100 units, respectively) with average modular structure length of 12 units to simulate difficult scenarios.

**Table 1:** WAMI running times

Task	Dataset	**Num**.	Iteration	Time
Alignment	MS205	91806	1	2.5
	MSY1	59340	1	3.25
	Random100	5000	1	0.3
	Random200	20000	1	1.3
	Random400	80000	1	5

Structural variation	MS205	91806	2	4
	MSY1	59340	2	6
	Random100	5000	2	0.6
	Random200	20000	2	2.3
	Random400	80000	2	9

	MSY1	59340	10	0.3
	MSY1	59340	25	1
	MSY1	59340	50	1.8
	MS205	91806	10	2.3
	MS205	91806	25	5.5
	MS205	91806	50	11
Directional bias	Random100	5000	10	0.2
	Random100	5000	25	0.5
	Random100	5000	50	1
	Random200	20000	10	1
	Random200	20000	25	2.1
	Random200	20000	50	4.1
	Random400	80000	10	3.5
	Random400	80000	25	8
	Random400	80000	50	16

Running times in minutes on WAMI for the MSY1 and MS205 (haplotype C) datasets. The column titled "Dataset" contains the dataset used. "Random100", "Random200", and "Random400" are datasets with 100, 200, and 400 artificial maps, respectively. The column titled "Num." contains the number of pairwise map alignments which need to be computed. The column titled "iteration" includes the number of randomization steps (and hence increased data size) in the analysis of duplication dynamics. The number of iterations in the task of analyzing structural variation is 2 because it runs one time on the original dataset and one time on randomized dataset of the same size.

**Figure 3:**
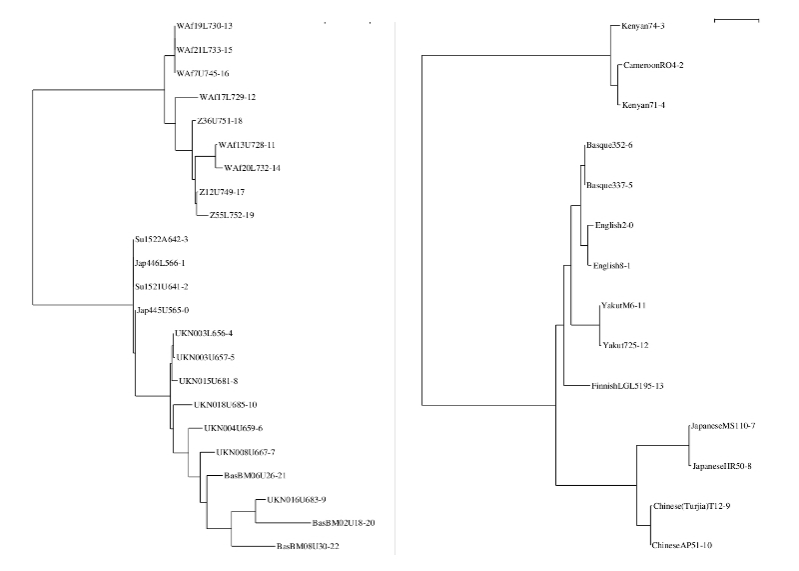
**Phylogenetic trees**. Left: Phylogenetic trees for a subset of the MS205 dataset, including individuals from different populations; Basque (BAS), UK, Surui (SU), Japanese (JAP), West African (Waf), and Zimbabweans (Z). (Here we use the original nomenclature distributed with the dataset.) Right: Phylogenetic tree for a subset of the MSY1 dataset including individuals from different populations. Individuals belonging to the same population are clustered together.

Figure 3 shows two phylogenetic trees produced by WAMI for a subset of the MS205 and MSY1 datasets. In these trees, we see that individuals from the same population are clustered together, which is in accordance with published results [2,3,6].

### Structural variation

**Figure 4:**
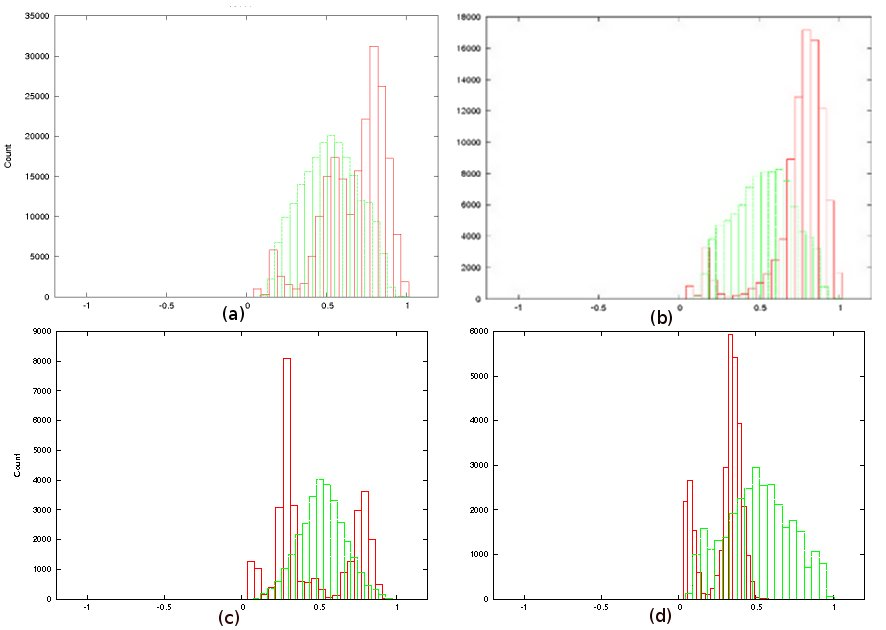
**Structural variations histograms**. (a): Histogram reported by WAMI for the MS205 dataset. The x-axis is the pivot-point normalized to the sequence lengths and the y-axis is its count. The red histogram is for the real dataset and the green histogram is for the randomized dataset. (See the original figures attached to the manuscript.) Accumulation at the right side indicates more variations at the 3' end. (b): Histogram for the subset of MS205 dataset including haplotype C. (c): Histogram of the MSY1 dataset, showing that the variations are bi-polar. (d): Histogram of MSY1, but after removing the units with Type 4 and 4a, showing that variations in this case accumulate only at the left side.

We applied WAMI to both datasets to investigate structural variation. When studying structure variation with MS205, Armour et al. [2,20] noticed polarized variability at the 3' end, where most of the differences between the alleles (individual maps) accumulate at the 3' end. Figure 4(a) and 4(b) shows the histograms of the pivot points obtained for the original MS205 dataset and a subset of it including haplotype C. It is clear that the histograms of the original datasets are biased to the right in comparison to that of a randomized datasets. This bias indicates polar variability towards the 3' end. These plots confirm the results obtained by Armour et al. [2]. (The presented results of MS205 are obtained using the double-copy option, but the results under the single-copy model are very similar.)

For the MSY1 dataset, lying on the Y chromosome, Jobling et al. [5] noticed high variability at the 5' end in contrast to the autosomal MS205 dataset, and they noticed also that Types 4 and 4a, existing almost solely at the 3' side, causes another source of variation at this end. This suggests bi-polar variability of this dataset. For us it was interesting to see how WAMI can thus help in spotting not only polar but also bi-polar variability. Figure 4(c) shows our observations for the MSY1 dataset. The resulting histogram has peaks at both ends. This indicates that the variations are bi-polar. To further verify our procedure on the MSY1 dataset, we removed Type 4 and 4a from the 3' end and repeated the experiment. Figure 4(d) shows biased histogram to the 5' end. That is, both extremities of the MSY1 maps are highly variable, and the unit types 4 and 4a already introduces another source of variation, verifying the observation of Jobling et al. [5].

### Duplication dynamics

**Figure 5:**
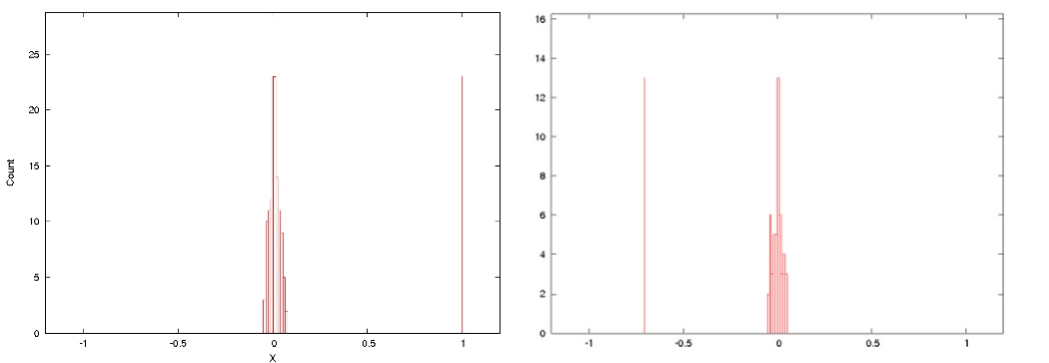
**Duplication dynamics histograms**. Left: Histogram to detect directional bias for the MSY1 dataset. The distribution of *E*_*n *_of the randomized data is centered around zero. The peak at point 1 on the x-axis is for *E*_*n *_of the original dataset, and it is clearly far from that of data with expected equal rates of left-to-right and right-to-left duplications. Right: Histogram to detect directional bias for the MS205 dataset. The peak on the left on the x-axis is for *E*_*n *_of the original dataset.

We used WAMI to study duplication dynamics with the MSY1 and MS205 datasets. Figure 5(left) shows the resulting histogram for MSY1. The peak value on the right shows *E*_*n *_of the real dataset, where *E*_*l *_= 876, and *E*_*r *_= 0. It is clear that this value is far from the *E*_*n *_values of the randomized datasets with expected equal rates of left-to-right and right-to-left duplications. That is, the plot indicates that left and right duplications do not contribute equally to the duplication history, and the units duplicate preferably in the direction 3' → 5', as conjectured by Jobling et al. [5]. In Figure 5 (right), we show the histogram for the MS205 dataset (haplotype C), which also shows directional bias, but this time towards the right ( *E*_*l *_= 1940, and *E*_*r *_= 13318). These results for both datasets may indicate the existence of unknown (chromosome-specific) dynamic constraints governing the duplication of the minisatellite units. Hence, they call for further investigation.

## Conclusions

In this paper, we presented WAMI, a web server for comprehensive analysis of minisatellites. The server provides many of the functionalities needed by researchers in this area. Future versions of the server are planned to provide data-mining functionalities for associating the map comparison results to other features, like age, ethnicity, or genetic markers on the chromosomes.

The algorithms of WAMI for minisatellite map analysis can also be used for comparing arrays for tandemly repeated units within proteins or gene sequences; the work of Rivals et al. [29] shows an example of this application. The alignment part of WAMI can also be used to compare parent/son *micro*satellite datasets, provided that the microsatellite units are mapped to symbols, in analogy to the unit typing step of minisatellites. In addition to its applications in parental tests, this comparison helps in studying the mutation rates in association with other map characteristics and helps in estimating parental ages. The work of Dupuy et al. [30] is an example of such studies.

In this paper, we rely on a map evolution model based on single- and double-copy duplications. In spite of the computational difficulty, it is still interesting to incorporate the multiple copy duplication model in map alignment, eventually through heuristic algorithms. Furthermore, it is also desirable to incorporate recently suggested evolutionary operations, such as boundary switch and modular structure change [31] appearing in some minisatellite datasets. These operations could be modeled by block exchange within the map, in an analogous way to the block exchange operation in genome rearrangement studies. But a practical solution to this problem is algorithmically challenging and remains a subject of future research.

## Availability and requirements

**Project name**: WAMI: A Web Server for the Analysis of Minisatellite Maps.

**Project home page**: http://www.nubios.nileu.edu.eg/tools/wami.

**Operating system(s)**: Platform independent (web server).

**Programming language**: Perl, C, Java script, JSF

**Other requirements**: Better viewed on the browsers FireFox, Internet Explorer 8 (IE8), Safari, and Opera. For local installation, Tomcat 6.0 or more, JDK 1.5 or more, Apache Ant 1.7 or more are needed.

**License**: Free for academics. Authorization license needed for commercial usage (Please contact the corresponding author for more details).

**Any restrictions to use by non-academics**: No restrictions.

## Authors' contributions

MA and RG contributed to theoretical developments which form the basis of WAMI. MA and MEK developed and tested the software. All authors wrote and approved the manuscript.
